# An efficient method for early Alzheimer’s disease detection based on MRI images using deep convolutional neural networks

**DOI:** 10.3389/frai.2025.1563016

**Published:** 2025-04-29

**Authors:** Samia Dardouri

**Affiliations:** ^1^Department of Computer Science, College of Computing and Information Technology, Shaqra University, Shaqraa, Saudi Arabia

**Keywords:** CNN, Alzheimer’s disease, deep learning, early detection, MRI

## Abstract

Alzheimer’s disease (AD) is a progressive, incurable neurological disorder that leads to a gradual decline in cognitive abilities. Early detection is vital for alleviating symptoms and improving patient quality of life. With a shortage of medical experts, automated diagnostic systems are increasingly crucial in healthcare, reducing the burden on providers and enhancing diagnostic accuracy. AD remains a global health challenge, requiring effective early detection strategies to prevent its progression and facilitate timely intervention. In this study, a deep convolutional neural network (CNN) architecture is proposed for AD classification. The model, consisting of 6,026,324 parameters, uses three distinct convolutional branches with varying lengths and kernel sizes to improve feature extraction. The OASIS dataset used includes 80,000 MRI images sourced from Kaggle, categorized into four classes: non-demented (67,200 images), very mild demented (13,700 images), mild demented (5,200 images), and moderate demented (488 images). To address the dataset imbalance, a data augmentation technique was applied. The proposed model achieved a remarkable 99.68% accuracy in distinguishing between the four stages of Alzheimer’s: Non-Dementia, Very Mild Dementia, Mild Dementia, and Moderate Dementia. This high accuracy highlights the model’s potential for real-time analysis and early diagnosis of AD, offering a promising tool for healthcare professionals.

## 1 Introduction

According to the Centers for Disease Control and Prevention (CDC) data, AD was the seventh leading cause of death in the United States in 2022 (Ahmad et al., 2023), while COVID-19 ranked fourth. AD is a neurodegenerative disorder characterized by progressive cognitive decline, memory loss, and behavioral changes. It is one of the most common causes of dementia among the elderly, affecting millions worldwide. Before the COVID-19 pandemic, AD was the sixth leading cause of death, following stroke. The shift in rankings highlights the significant impact of the pandemic on public health, while AD continues to be a major concern due to its progressive nature and the aging population. AD is the leading cause of dementia, accounting for 60–80% of dementia cases (Kumar et al., 2018). This neurodegenerative form of dementia typically begins with mild cognitive impairment (MCI) and progressively worsens, impacting brain cells, causing memory loss, affecting thinking skills, and hindering the performance of simple tasks. As a result, AD is characterized as a progressive and multifaceted neurological brain disease. Individuals with MCI have a higher likelihood of developing AD compared to others.

The effects of AD become noticeable only after years of changes in the brain, as the disease initiates two decades or more before symptoms are detected. AD International (ADI) reports that over 50 million people worldwide are currently grappling with dementia, with projections estimating a rise to 152 million people by 2050. This implies that every 3 s, someone develops dementia (Alzheimer’s Association, 2022).

Early diagnosis is crucial in slowing the progression of the disease, offering potential for better treatment outcomes and improved quality of life for patients. However, diagnosing AD remains a significant challenge due to the overlapping symptoms with other forms of dementia and the subtle, gradual onset of its early stages. Alzheimer’s experts face a significant challenge due to the lack of a reliable treatment for the disease. Although existing therapies can mitigate symptoms, there’s no cure. Hence, early detection during the prodromal stage is vital. Computer-Aided Systems (CAD) play a crucial role in accurately detecting AD early on, aiming to mitigate the anticipated rise in care costs. Magnetic Resonance Imaging (MRI) plays a pivotal role in the detection of structural brain changes associated with AD.

MRI scans provide detailed images of the brain, enabling clinicians and researchers to study the atrophy patterns in brain regions, such as the hippocampus, which are critical indicators of Alzheimer’s progression. While manual interpretation of MRI scans by experts remains a standard practice, it is time-consuming, subjective, and prone to variability between clinicians. To address these limitations, automated approaches based on artificial intelligence (AI), particularly deep learning, have been increasingly explored for AD detection. Traditional Machine Learning (ML) techniques rely on manual feature extraction, which is time-consuming and subjective. Deep learning (DL), particularly convolutional neural networks (CNNs), offers a solution by automatically extracting features, thus improving efficiency (Kim et al., 2022; Rahim et al., 2023; EL-Geneedy et al., 2023).

This study is dedicated to diagnosing and categorizing AD using an image dataset. Despite the subtle symptoms of AD, there remains a pressing need to address the identified research challenges. The study contributes to the field of AD research in several key ways. It offers a solution for precise and timely diagnosis of AD at an early stage. While the cause of the disease remains largely unknown, except for rare familial cases linked to genetic mutations, this study focuses on addressing the diagnostic aspect. These architectures are utilized to classify images into normal or abnormal (AD) categories, encompassing the four different stages of AD. It also implements binary medical image classifications between each pair of AD stages, using CNN architecture (Al Shehri, 2022; Vrahatis et al., 2023).

## 2 Related works

Research is a continuous journey where researchers meticulously analyze raw data from various angles, employing diverse methodologies and mechanisms. Through this process, they aim to draw profound insights from the data, ultimately constructing decision support models that assist decision-makers across various domains in their roles. Similarly, the detection of AD has emerged as a focal point of recent research endeavors, prompting the exploration of various methodologies, with ML and DL emerging as common approaches for automatic detection (Helaly et al., 2022). Given the emphasis of this study on DL methodology, our discussion will be confined to DL models found in the existing literature.

Several reviews, particularly from Fathi et al. (2022), Singh et al. (2024), and Malik et al. (2024), provide an extensive overview of the current advancements and challenges in applying deep learning and ML for AD detection and prediction, particularly focusing on early diagnosis and disease progression. Across these studies, CNNs are consistently found to outperform traditional ML approaches, showcasing their potential in improving diagnostic accuracy and aiding in the treatment of neurological diseases. Singh et al. (2024) utilized VGG19 pre-trained model is fine-tuned and achieved an accuracy of 97% for multi-class AD stage classifications.

Additionally, some researchers have focused on unsupervised feature learning, extracting features from raw data using methods like scattered filtering and neural networks, followed by classification with sparse filtering and regression techniques. Alsubaie et al. (2024) provides a systematic review of deep learning approaches applied to neuroimaging for AD detection. It examines different deep learning models such as CNNs, autoencoders, and recurrent neural networks, and evaluates their performance across a variety of datasets, including MRI and PET scans. Diogo et al. (2022) developed a multi-diagnostic system that integrates data from various sources and showed that their model outperformed traditional diagnostic methods. Previous studies (Aderghal et al., 2020; Bae et al., 2020) explored transfer learning techniques applied to CNNs for Alzheimer’s disease (AD) classification. Specifically, Aderghal et al. (2020) investigated the use of multiple MRI modalities to enhance AD stage categorization, while Bae et al. (2020) focused on detecting Alzheimer’s using T1-weighted MRI scans with a CNN-based model. The study highlights the significance of integrating data from different sources to improve diagnostic accuracy and generalizability. The previous study (Dyrba et al., 2021) proposes a novel approach to enhance the interpretability of 3D CNN models used for AD detection. The authors introduced relevance maps that visualize the regions in MRI images contributing most to the model’s predictions. This interactive visualization helps improve the transparency of the decision-making process in deep learning models. The study evaluated the performance of the model on Alzheimer’s datasets and showed how visualization tools can improve the usability of CNNs in clinical settings by making the outputs more interpretable for clinicians. Liu et al. (2021) introduces the use of depth wise separable convolutional neural networks (DSCNNs) for AD detection. DSCNNs are a variation of traditional CNNs designed to reduce model complexity and computational cost while maintaining performance (Qasim Abbas et al., 2023).

In previous study, Lien et al. (2023) investigates the use of convolutional neural networks (CNNs) to classify the severity of AD based on SPECT (single-photon emission computed tomography) images. The study compares different CNN architectures and evaluates their performance in detecting mild cognitive impairment (MCI) and AD stages. The authors found that deep learning models could classify AD severity with a high degree of accuracy, outperforming traditional ML techniques.

In previous research (Nithya et al., 2023; Mehmood et al., 2022; Şener et al., 2024; Mora-Rubio et al., 2023; Singh et al., 2023; George et al., 2023) presents an efficient 3D CNN framework enhanced by attention mechanisms for classifying AD. The proposed model leverages 3D convolutional neural networks to capture spatial and temporal features from volumetric MRI scans, improving the model’s ability to discern subtle differences associated with varying stages of the disease. By utilizing 3D data from MRI scans, the model captures spatial and volumetric features associated with Alzheimer’s progression. The study shows that 3D-CNNs can significantly improve diagnostic accuracy compared to 2D-CNNs, particularly in detecting subtle brain changes that occur in the early stages of the disease.

Deep learning (DL) has emerged as a powerful methodology in the field of diagnostic imaging, as highlighted by several recent studies. However, diagnosing AD using DL remains a significant challenge for researchers. Issues such as the scarcity and lower quality of medical images, difficulties in identifying regions of interest (ROIs) within the brain, and class imbalances complicate AD detection (Şener et al., 2024). Among various DL architectures, convolutional neural networks (CNNs) have garnered considerable attention due to their exceptional effectiveness in classification tasks. Unlike conventional ML approaches, deep learning facilitates automatic feature extraction, capturing representations from low-level to high-level features (Mora-Rubio et al., 2023). As a result, deep learning methods require minimal image preprocessing and less prior knowledge about the synthesis process. Data size plays a crucial role, as a larger dataset facilitates better learning of model parameters and improves generalization by effectively capturing intrinsic data characteristics. However, a significant challenge in applying DNN to AD detection and diagnosis is the limited availability of large training datasets for learning discriminative patterns in high-dimensional feature spaces. This limitation arises because a larger dataset helps prevent overfitting issues that may occur in deep-learning models (Singh et al., 2023).

Imbalanced datasets present a significant challenge in medical disease detection, particularly for AD, where the number of samples in each class is often unequal. This imbalance can bias model performance, making generalizations difficult. While individual deep learning models can handle basic data efficiently, they may struggle with more complex problems, leading to overfitting and poor generalizability. Such models typically operate with a single set of weights and may fail to capture the nuanced features of the images. For accurate disease diagnosis using segmented MRI, an in-depth examination of disease-specific tissues is essential. Although several studies have employed conventional machine-learning methods for MRI diagnostics (George et al., 2023; Ahmed et al., 2023; Manu et al., 2022), these approaches often rely on manually derived features and necessitate extensive medical staff involvement. This complexity makes the conventional methods time-consuming and prone to errors, resulting in imprecise diagnoses and inefficiencies in the diagnostic process.

Venkatasubramanian et al. (2023) introduces a hybrid approach for AD prediction using a pretrained Convolutional Neural Network (CNN) model optimized with a Discrete Harris Hawks Optimization (DHO) algorithm. The study aims to improve the accuracy and efficiency of AD detection from MRI images. The pretrained CNN model is fine-tuned with the DHO algorithm to optimize hyperparameters, enhancing the predictive capabilities of the model. The model was tested on MRI datasets, and the results demonstrated superior performance compared to other traditional CNN models, offering a robust tool for the early detection of AD.

In previous study, Alorf and Khan (2022) focuses on multi-label classification of AD stages using resting-state functional MRI (fMRI) data and deep learning techniques. The authors developed a model that utilizes correlation connectivity data from fMRI scans to classify patients into different stages of AD. The multi-label classification approach enables the detection of overlapping symptoms and disease progression stages, which are often challenging to differentiate. The proposed deep learning framework demonstrates high accuracy in classifying multiple AD stages, offering a potential tool for improving early diagnosis and personalized treatment plans. Another innovative method (Swarnalatha and Kaur, 2023) presents a greedy optimized intelligent framework designed for the early detection of AD using electroencephalogram (EEG) signals. The framework aims to improve diagnostic accuracy by leveraging advanced signal processing techniques and ML algorithms. Tajammal et al. (2023) introduces a deep learning-based assembling technique aimed at classifying stages of AD using functional MRI (fMRI) data. For multiclass classification of AD, the results of VGG-16, ResNet-18, AlexNet, Inception V1, and Custom CNN are combined. The results show that the max-vote assembling technique achieves 98.8% accuracy.

Mujahid et al. (2023) introduced an efficient ensemble approach for detecting AD, combining deep learning techniques with an adaptive synthetic oversampling method to address the challenges posed by imbalanced datasets. The proposed framework utilizes multiple deep learning models, creating an ensemble that leverages their strengths for improved classification accuracy 97.35%.

Mandal and Mahto (2023) proposes a novel deep learning approach using a Multi-Branch Convolutional Neural Network (CNN) for the early detection of AD from brain MRI images. The primary goal is to improve diagnostic accuracy and enable early intervention. The model leverages MRI scans and classifies them into different stages of AD.

El-Assy et al. (2024) presents a novel Convolutional Neural Network (CNN) architecture designed for the early detection and classification of AD using MRI data. The study achieved a significant improvement in diagnostic accuracy compared to previous models, utilizing advanced deep learning techniques. The CNN model was trained on a large dataset of MRI scans, classifying patients into various stages of AD progression. The authors emphasize the potential of this method to aid in the early diagnosis of AD, offering an effective tool for healthcare professionals.

In previous study, Wang (2024) develops deep neural networks (DNNs) aimed at diagnosing AD and dementia from MRI images. The approach focuses on early-stage detection, incorporating advanced neural network architectures to analyze brain imaging data and classify the disease. The study highlights the use of transfer learning and data augmentation techniques to enhance the model’s generalizability, addressing the challenges of limited training data. This method shows promise in improving diagnostic accuracy, particularly for early intervention in AD. Liu et al. (2022) propose a deep learning model for early detection of AD that focuses on generalizability across different patient populations. This model was trained using structural MRI scans and tested on various datasets to ensure it could generalize well to new data. The study demonstrates that the model performs reliably across different MRI scanners and datasets, making it a robust tool for real-world applications in AD detection. The results indicate that the model can aid in the early diagnosis of AD, even in diverse clinical settings.

This research focuses on diagnosing and classifying AD using an image dataset. In this research, we aimed to advance the detection of AD using deep learning techniques. Specifically, we focused on addressing key limitations in existing methodologies by developing a novel deep convolutional neural network (CNN) architecture and ADAM optimizer. This architecture features a high parameter count of 6,026,324 and is designed with three distinct convolutional branches, each varying in length and using different kernel sizes. This improves the model’s precision and robustness, leading to more accurate and reliable detection of Alzheimer’s disease (AD). The novelty of this study lies in the unique architecture and comprehensive evaluation of the CNN model, aiming to significantly improve detection accuracy and overall performance in diagnosing AD.

## 3 Materials and methods

### 3.1 Description of Alzheimer’s MRI dataset

**Figure 1:**
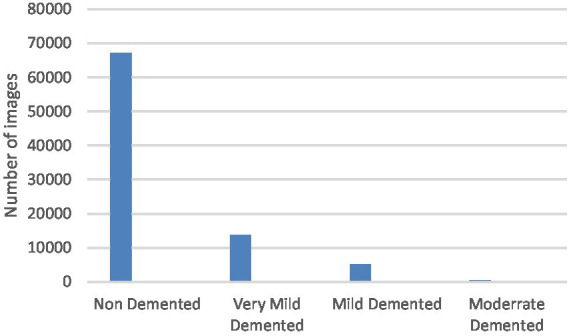
Class distribution of the MRI dataset.

The Dataset is consisting of preprocessed MRI (Magnetic Resonance Imaging) Images. The brain images were sliced along the z-axis into 256 pieces, and slices ranging from 100 to 160 were selected from each patient. The dataset (Kaggle, 2021) includes 80.000 different MRIs (Magnetic Resonance Image) collected from different sources were given in this paper. Patient classification was performed based on the provided metadata and Clinical Dementia Rating (CDR) values, resulting in four classes belong to 4 different classes as seen in Figure 1. These classes are as follows; Mild Demented, Moderate Demented, Non Demented and Very Mild Demented. We selected this dataset for its numerous advantages: it is freely accessible, provides a variety of diagnostic classifications, and requires minimal storage capacity, making it distinct from other commonly used datasets in the field. For standardization, the MRI scans were resized to a uniform resolution of 100 × 100 pixels.

**Figure 2:**
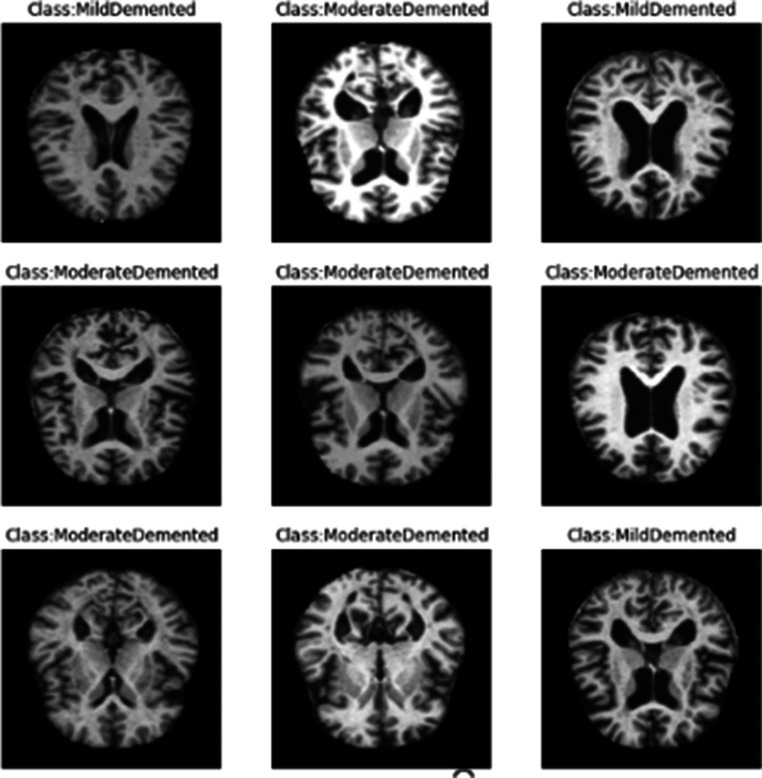
Samples of dataset images.

The entire dataset is utilized for both training and testing, maintaining a ratio of 70% for training and 30% for testing. Figure 2 illustrates sample images from the dataset.

### 3.2 Proposed methodology

The main goal of this study was to develop an effective and robust neural network model for the early diagnosis of AD. To accomplish this, we designed a multi-branch convolutional neural network (CNN) using Keras (2017), which is seamlessly integrated with TensorFlow (2022). The approach comprises several key steps:

#### 3.2.1 Input and preprocessing

Initially, the brain MRI image is used as the input. The first step in preprocessing involves data normalization to ensure uniformity across the images. This process includes image thresholding to remove background noise, followed by dilations to eliminate small noise regions. After reducing noise, the images undergo further normalization to standardize intensity values, setting the mean intensity value to zero and the standard deviation to one. This normalization step is essential for stabilizing the training process and ensuring that the input data is consistently formatted, which enhances the model’s performance. Preprocessing is a crucial aspect of the AD detection system, involving essential steps to enhance the quality and reliability of data extracted from brain scans. This section focuses on the key preprocessing techniques utilized to prepare acquired imaging data before further analysis and interpretation. One of the initial preprocessing steps involves image registration, wherein brain scans are aligned to a common reference space. This alignment compensates for variations in positioning and orientation, ensuring consistent analyses across different individuals and time points. Common techniques for image registration include affine and non-linear transformations. Another critical preprocessing step is noise reduction, aimed at minimizing unwanted artifacts and noise that may interfere with subsequent analyses. Techniques such as Gaussian filtering and wavelet denoising are commonly employed to reduce noise while preserving essential features in brain images. Spatial smoothing is an additional preprocessing technique involving the application of a smoothing filter to the data. This process reduces local variations and enhances the signal-to-noise ratio, facilitating the identification of relevant patterns and structures in brain scans. Furthermore, motion correction is performed to address motion-related artifacts that may occur during brain scan acquisition. Motion correction algorithms can detect and rectify head movements, ensuring that the data accurately represent the structural and functional characteristics of the brain. It is essential to recognize that preprocessing techniques may vary depending on the imaging modality used, such as MRI. Each modality may necessitate specific preprocessing steps tailored to its unique characteristics and challenges.

#### 3.2.2 Stratified K-fold cross-validation

In the case of dataset imbalance, where certain classes are underrepresented compared to others, Stratified K-Fold Cross-Validation is an excellent technique to ensure that the class distribution is preserved across all folds. Stratified K-Fold Cross-Validation divides the dataset into k folds, just like regular K-Fold Cross-Validation, but it ensures that each fold contains approximately the same proportion of each class. This prevents the model from being biased toward the majority class, which can be a common issue in imbalanced datasets.

#### 3.2.3 Data augmentation

**Figure 3:**
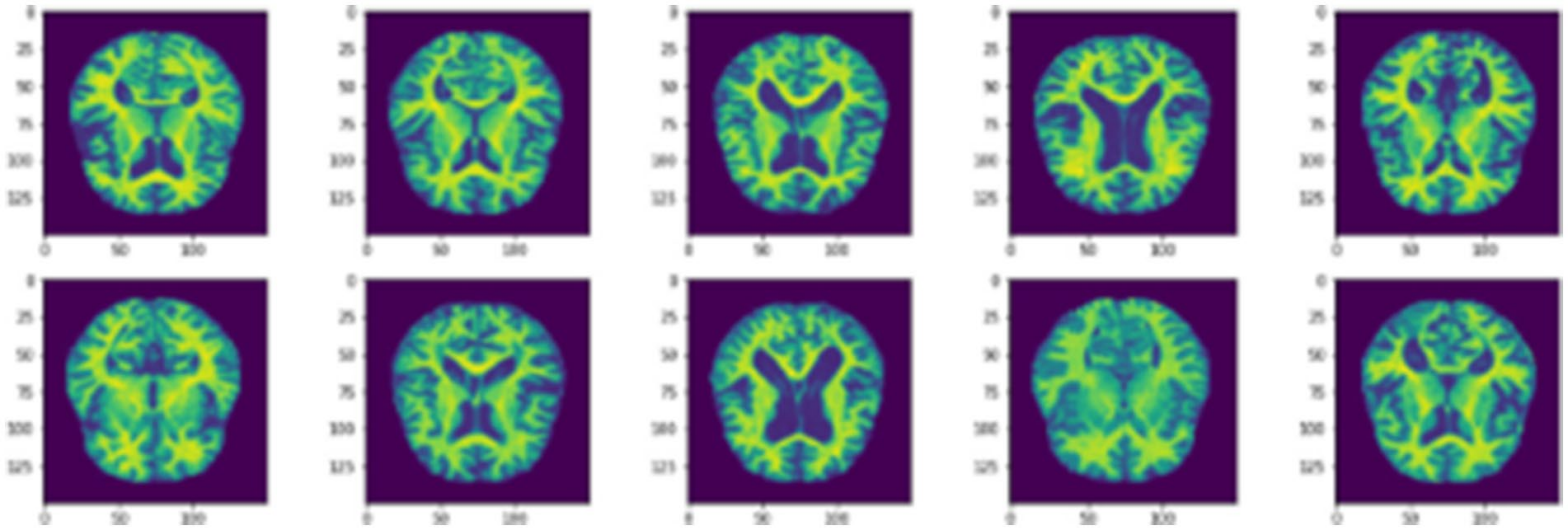
Samples of data augmentation images.

To address the imbalance in the collected dataset, which is evident in Figure 1, class balancing becomes necessary to enhance the classifier’s understanding of minority classes. While creating a large, benchmarked, medically tested image set is challenging, this obstacle can be overcome through data augmentation techniques. Data augmentation involves introducing random variations to existing training images while preserving class labels. Unlike contingency simulation, which is burdensome and error-prone, data augmentation offers a low-cost and effective means of increasing the diversity and representativeness of training data. In this paper, data augmentation was employed using various techniques such as rotation, shearing, zooming, and horizontal and vertical flipping. As a result, the number of images increased to a total of 84,074 as depicted in Figure 3.

#### 3.2.4 Model building

The proposed model is built upon the CNN architectures, both of which are widely used in deep learning for feature classification tasks. CNN (Convolutional Neural Network) models are designed to automatically and efficiently recognize patterns in images. CNNs are currently the most advanced technique used to detect of AD in medical images. In this process, the CNN model relies solely on MRI scans and does not consider the corresponding masks. This means that the CNN analyzes the MRI images alone to make predictions about the presence or characteristics of tumors, without taking into account any additional information provided by the masks. These masks may contain segmentation or labeling data related to tumor regions. The initial layer of a CNN is typically a Convo layer, which uses filters to extract features from the images. The output of this layer is a set of feature maps that indicate how each filter reacts to the input image. The second layer, known as the pooling layer, is usually added to reduce the size of the feature map while preserving important features. This helps in reducing the number of parameters and prevents overfitting. The output from both layers is then compressed and passed through fully connected layers, which ultimately classify the extracted features. The choice of our custom CNN architecture with three distinct convolutional branches was motivated by the need to capture multi-scale spatial features in MRI scans, enhancing the model’s ability to distinguish subtle differences across Alzheimer’s disease stages. Unlike traditional architectures such as VGG or ResNet, which follow a sequential deep structure, our design allows the model to extract fine-grained local features (with smaller kernels) while simultaneously capturing broader contextual patterns (with larger kernels).

**Figure 4:**
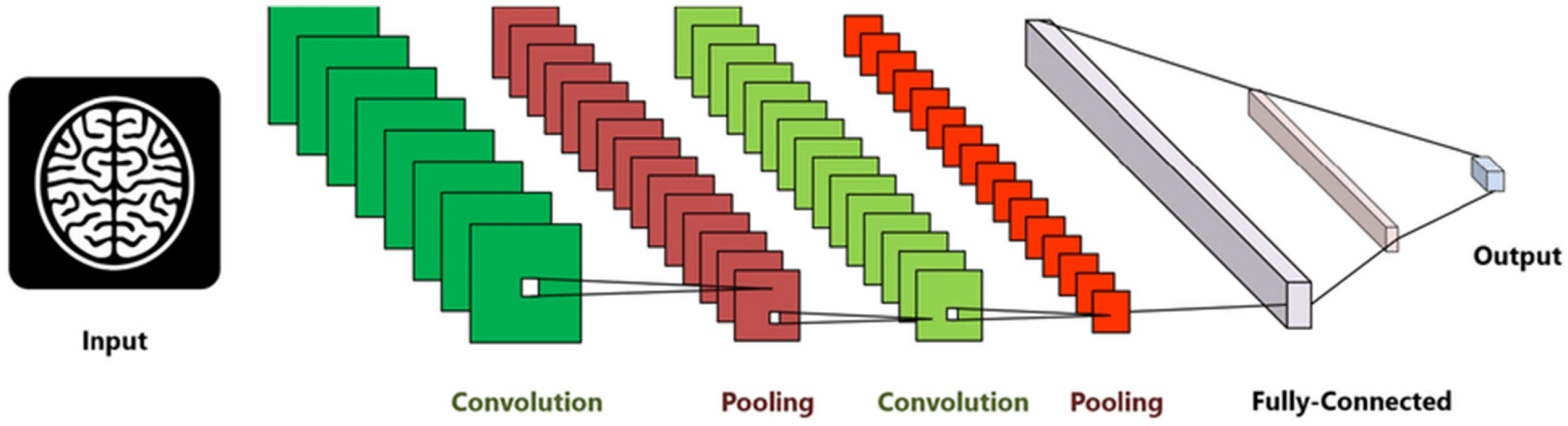
Convolution neural network architecture used for AD diagnosis (Abdul Azeem et al., 2021).

CNN is a type of neural network designed to process data with a grid structure. The convolution layer, which is based on linear algebra operations, is essential in this network. Another commonly used layer is the pooling layer, which can take the maximum or average value of pixel portions in an image. CNN can learn complex features by creating feature maps through the use of convolution layer kernels. These feature maps are then processed by the max-pooling layer to preserve relevant features and discard others. The fully connected layer converts the features into a one-dimensional vector for calculating output probability. The overall configuration of CNN is depicted in Figure 4.

The model, comprising 6,026,324 parameters, incorporates three distinct convolutional branches with varying lengths and kernel sizes to enhance feature extraction. Each branch is designed to process the input data at different scales, enabling the model to capture both fine-grained details and broader patterns effectively. Smaller kernels focus on localized features such as edges and textures, while larger kernels detect more global structures like shapes and contours. By varying the lengths of these branches, the network combines shallow layers for basic feature detection with deeper layers for learning complex, high-level abstractions. This multi-branch architecture enriches the feature representation and ensures comprehensive analysis, making the model well-suited for tasks requiring multi-scale feature understanding.

#### 3.2.5 Model training with ADAM optimizer

The model is trained using the ADAM optimization algorithm, which is specifically designed for deep neural networks. ADAM combines the benefits of the Adaptive Gradient Algorithm (AdaGrad) and Root Mean Square Propagation (RMSProp), computing adaptive learning rates for each parameter to enhance training efficiency and convergence. ADAM adapts the learning rate for each parameter based on the first-order moment (mean) and second-order moment (uncentered variance) of the gradients. This feature ensures parameters with sparse gradients are updated differently from those with dense gradients, leading to more efficient training.

#### 3.2.6 Evaluation

The model’s performance is assessed using several metrics, including precision, recall, sensitivity, specificity, accuracy, and F1-score. These metrics provide a well-rounded evaluation of the model’s ability to detect brain tumors from MRI images. To ensure robustness and generalization, the model is tested on a separate dataset, demonstrating its effectiveness in real-world scenarios.

**Figure 5:**
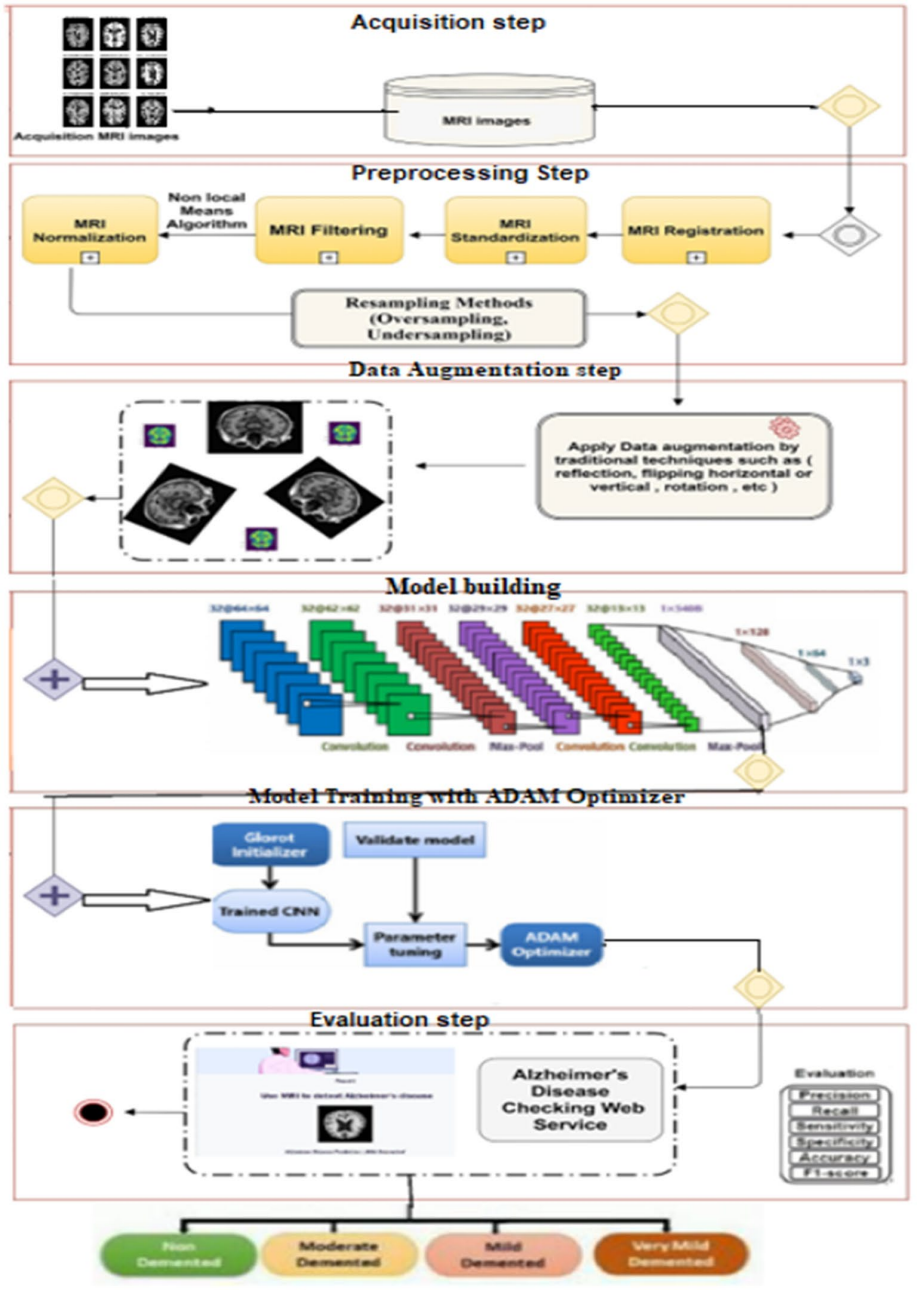
Proposed model methodology.

Figure 5 briefly represents the workflow of the proposed approach:

Alzheimer’s disease, an incurable neurodegenerative condition primarily affecting memory in the elderly, poses significant challenges in diagnosis due to its prevalence. Manual diagnosis is impractical given the large number of patients, and health specialists may make errors due to time constraints and the complexity of the evaluation process. While various procedures exist for Alzheimer’s diagnosis, there is a pressing need for an accurate and timely diagnostic solution. The figure illustrates the research methodology employed in this study to achieve an accurate and early diagnosis of AD. This proposed methodology directly tackles the issues outlined in the Introduction. While previous discussions have covered various techniques based on deep learning, they have fallen short in addressing the challenge of early AD diagnosis, particularly when symptoms are subtle or absent. This research study specifically concentrates on CNN-based deep learning model renowned for their effectiveness in diagnosing and classifying AD. Subsequently, Deep Convolutional Neural Networks (CNNs) are employed, and we advocate the utilization of the ADAM optimization function to construct predictive models based on the normalized dataset. This suggested approach holds promise for real-time analysis and classification of AD. Future plans involve expanding disease detection with additional datasets and employing different measures to enhance the system’s accuracy.

## 4 Experimental results and discussion

The model was trained on an NVIDIA RTX 3090 GPU with 16GB RAM using TensorFlow 2.9 in a Windows 11 environment. The training process utilized a batch size of 32, a learning rate of 0.001, and the Adam optimizer, which was chosen for its efficiency in deep CNN training.

### 4.1 Evaluation metrics

The proposed approach is evaluated using various performance metrics, including Recall, Precision and Accuracy presented in Table 1. These criteria are used to measure the classification efficiency of the improved CNN. In this table, “FN” represents false negatives, “FP” represents false positives, “TN” represents true negatives, and “TP” represents true positives.

**Table 1:** Evaluation metrics.

Technique	Formula
Accuracy	(TP+TN)/(TP+FP+TN+FN)
Recall	TP/(TP+FN)
Precision	TP/(TP+TN)

#### 4.1.1 Matthews correlation coefficient (MCC)

MCC is a performance metric that measures the correlation between the true and predicted classifications. It takes into account all four categories of the confusion matrix: true positives, true negatives, false positives, and false negatives. MCC is computed using the following formula:


$$MCC=\frac{\left(TP*TN\right)-\left(FP*FN\right)}{\sqrt{\left(TP+FP\right)\left(TP+FN\right)\left(TN+FP\right)\left(TN+FN\right)}}$$

#### 4.1.2 Cohen’s Kappa

Cohen’s Kappa measures the level of agreement between the true and predicted classifications while accounting for the possibility of agreement occurring by chance. Cohen’s Kappa (*κ*\kappaκ) is calculated as:


$$k=\frac{P_{0}-P_{e}}{1-P_{e}}$$

Where:

$P_{0}$ : Observed agreement (accuracy).

$P_{e}$ : Expected agreement by chance, given by:


$$P_{e}=\frac{\left(TP+FP\right)\left(TP+FN\right)+\left(FN+TN\right)\left(FP+TN\right)}{\mathrm{Tota}l^{2}}$$

In our model, the Cohen’s Kappa score of 70.09% and Matthews Correlation Coefficient (MCC) of 77.68% demonstrate strong classification performance. The MCC value indicates a high correlation between predicted and actual classifications, ensuring balanced performance across all classes. Meanwhile, the Cohen’s Kappa score accounts for agreement occurring by chance, confirming substantial reliability in the model’s predictions. These metrics validate the effectiveness of our approach in pneumonia detection, reinforcing its potential for real-world medical applications while highlighting opportunities for further optimization.

### 4.2 Discussion

**Figure 6:**
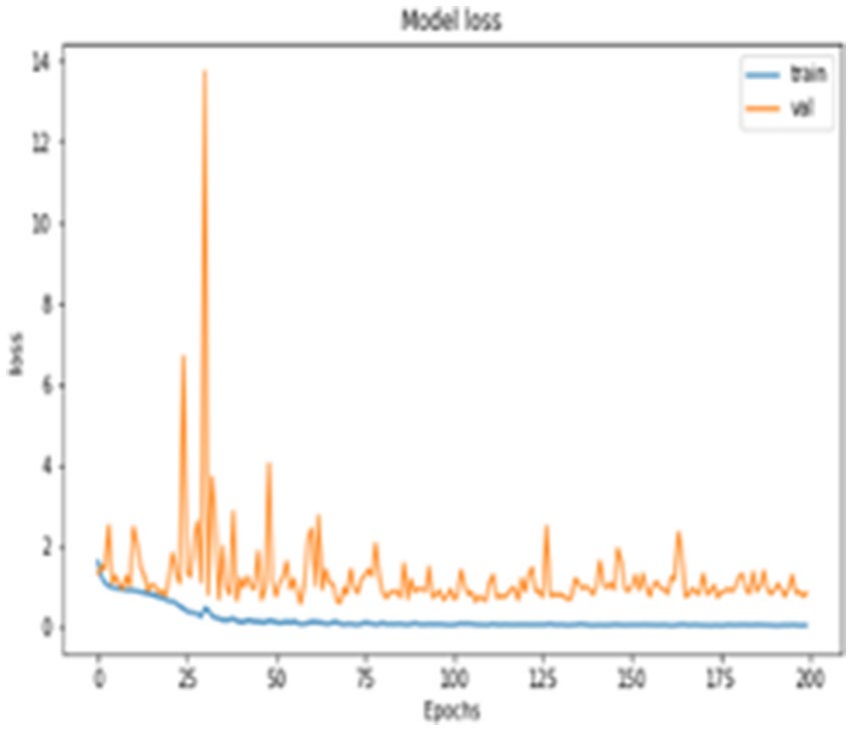
Model loss.

In addition to quantitative metrics, the performance of the network is qualitatively assessed through loss graph plotted against the number of epochs. During the training phase, the loss values for both training and testing, as well as the loss of validation data, were measured, as depicted in Figure 6. It appears that you are describing the role of validation loss in your model’s performance over training epochs, highlighting its reduction as training progresses. The description of Figure 6 emphasizes that the model’s validation loss decreased notably by the 200th epoch, showcasing significant performance improvement over time.

**Figure 7:**
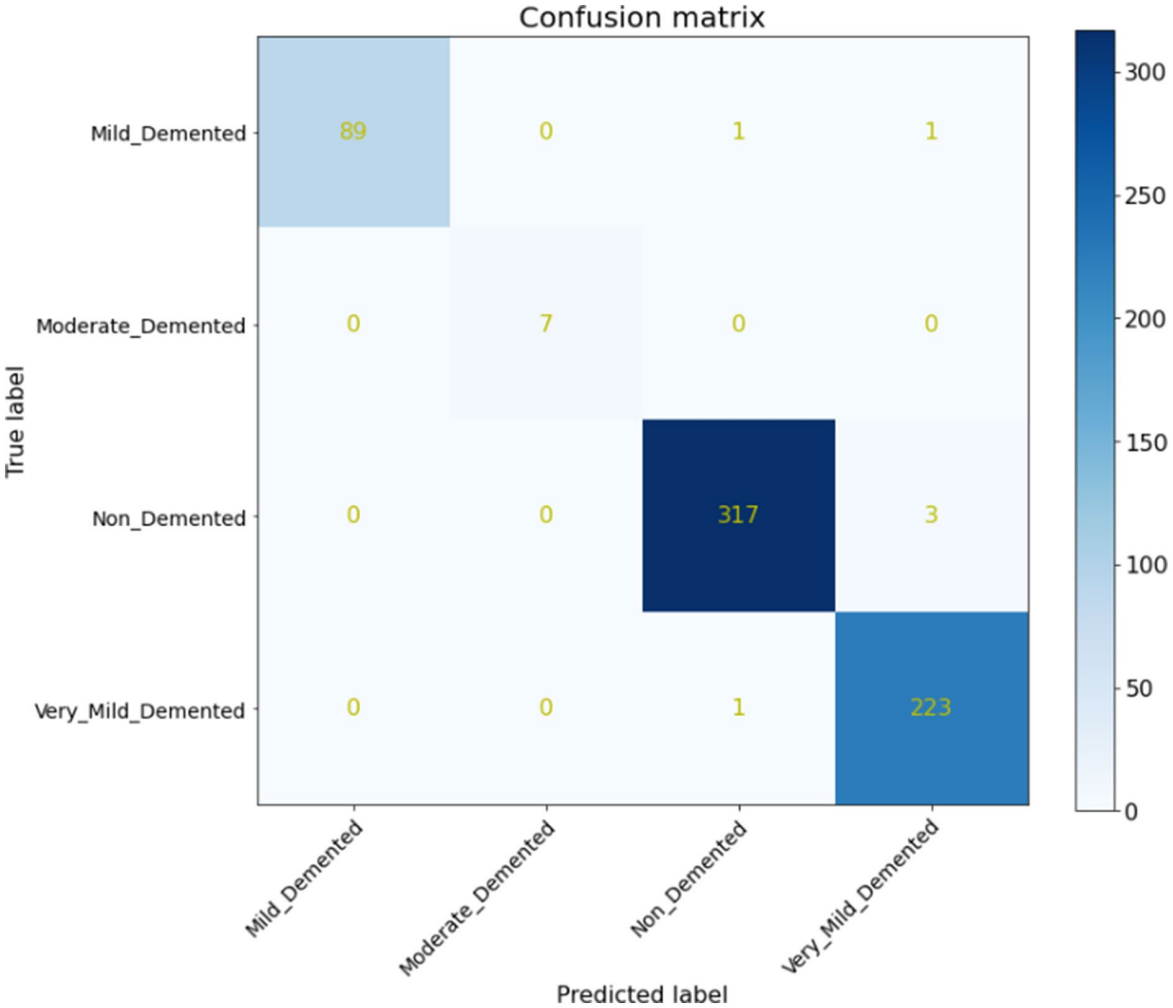
Confusion matrix.

Furthermore, a quantitative evaluation is conducted using a confusion matrix, shown in Figure 7. The confusion matrix is valuable not only for assessing which classes may be misclassified more frequently but also for model evaluation, monitoring, and management. It provides insights into the performance of the proposed system, aiding in the identification of any specific classes that may be challenging for the model to accurately classify. Your neural network’s performance is remarkable, achieving an accuracy of 99.68%, as illustrated by the confusion matrix in Figure 7. Out of 642 test images, only 6 were misclassified, with the misclassifications occurring between the non-demented, mild demented and very mild demented classes. This suggests that while the model performs exceptionally well overall, the fine distinction between these two stages could still be a challenge due to the subtle differences between them.

As indicated in Table 2, our model attained the highest accuracy rate of 99.68% and the average F1-Score of 99.25%, along with an average precision of 99.5%.

**Table 2:** Performance of proposed model.

Technique	Precision	Recall	F1-Score
Mild demented	0.99	0.97	0.98
Moderate demented	1.00	1.00	1.00
Non demented	0.99	1.00	1.00
Very mild demented	1.00	0.99	0.99

As Table 3 illustrates, the proposed technique outperformed its peers in terms of accuracy, representing a significant breakthrough that could reshape the field of AD diagnosis. This advancement offers hope for more accurate and early diagnoses, which can lead to better patient outcomes and a deeper understanding of the disease’s progression. The thorough comparison emphasizes the importance of this innovation, positioning the proposed technique as a leading approach in AI-assisted AD diagnosis using MRI images.

**Table 3:** Comparative evaluation of AI-enhanced AD diagnosis using MRI images: proposed technique vs. previous models.

References	Dataset	Number of images for each classes	Technique	Accuracy rate
Al Shehri (2022)	5,000 images from Kaggle	Non-Demented (Healthy Control - HC): ~2500 images Mild Cognitive Impairment (MCI): ~1500 images Alzheimer’s Disease (AD): ~1000 images	DenseNet-169	97.7%
Helaly et al. (2022)	ADNI	Healthy Control (HC): ~2000+ images Mild Cognitive Impairment (MCI): ~3000+ images Alzheimer’s Disease (AD): ~2000+ images	VGG-19	97%
Dyrba et al. (2021)	ADNI	Healthy Control (HC): ~2000+ images Mild Cognitive Impairment (MCI): ~3000+ images Alzheimer’s Disease (AD): ~2000+ images	ResNet-50	88.7%
			3D CNN	91%
Liu et al. (2021), Nithya et al. (2023), Mujahid et al. (2023)	OASIS	Total Scans: ~7,000 MRI scans Non-Demented (CDR = 0): Majority (~70%) Very Mild Demented (CDR = 0.5): ~20% Mild to Moderate Demented (CDR = 1 or 2): ~10%	AlexNet with transfer learning	91.40%
			GoogLeNet with transfer learning	93.02%
	ADNI	Healthy Control (HC): ~2000+ images Mild Cognitive Impairment (MCI): ~3000+ images Alzheimer’s Disease (AD): ~2000+ images	3D CNN	89.47%
	5000 images from Kaggle	Non-Demented (Healthy Control - HC): ~2500 images Mild Cognitive Impairment (MCI): ~1500 images Alzheimer’s Disease (AD): ~1,000 images	CNN	97.35%
Proposed model	OASIS	Total Scans: ~7000 MRI scans Non-Demented (CDR = 0): Majority (~70%) Very Mild Demented (CDR = 0.5): ~20% Mild to Moderate Demented (CDR = 1 or 2): ~10%	CNN+Adam optimizer	99.68%

## 5 Conclusion and future work

Manual diagnosis is impractical given the large number of patients, and health specialists may make errors due to time constraints and the complexity of the evaluation process. While various procedures exist for Alzheimer’s diagnosis, there is a pressing need for an accurate and timely diagnostic solution. The proposed model introduces a deep learning-based method utilizing the CNN architectures for diagnosing and classifying AD. The model categorizes Alzheimer’s into four classifications: Non-Dementia, Very Mild-Dementia, Mild Dementia, and Moderate Dementia. This suggested approach holds promise for real-time analysis and classification of AD.

The study acknowledges several limitations in its current approach. First, only one data source included patients with mild cognitive impairment (MCI), which restricts the comprehensiveness of the model. Additionally, the testing set does not fully mimic real-world clinical situations faced by physicians, limiting its direct applicability to clinical decision-making. For future development, the study emphasizes the importance of thoroughly evaluating the classifier in real-world clinical settings, using a larger, longitudinal, and multi-site dataset. This would allow for a better understanding of how the algorithm could impact patient care. The paper suggests that future studies should also assess clinicians’ performance with and without the algorithm’s assistance to gauge its effectiveness. To enhance robustness, future work will focus on evaluating the model using independent datasets such as ADNI and AIBL, which contain diverse MRI scans from different sources. This will help assess the model’s ability to generalize across varying imaging protocols and population demographics. Additionally, domain adaptation techniques and transfer learning strategies could be explored to improve performance when applied to unseen datasets. These enhancements will ensure a more comprehensive validation of the proposed approach and strengthen its clinical applicability.

## Data Availability

The original contributions presented in the study are included in the article/supplementary material, further inquiries can be directed to the corresponding author.
